# Auger Electron Spectroscopy for Chemical Analysis of Passivated (Al,Ga)N-Based Systems

**DOI:** 10.3390/mi17010047

**Published:** 2025-12-30

**Authors:** Alina Domanowska, Bogusława Adamowicz

**Affiliations:** Institute of Physics—CSE, Silesian University of Technology, S. Konarskiego 22B, 44-100 Gliwice, Poland

**Keywords:** gallium nitride, passivation, characterization, Auger electron spectroscopy, depth profiling, dielectric/semiconductor interface

## Abstract

This review summarizes the use of Auger Electron Spectroscopy (AES) for microchemical analysis of two different types of dielectric/(Al,Ga)N-based systems: (i) extrinsic dielectric PECVD SiO_2_, ALD Al_2_O_3_, and ECR-CVD SiN_x_ films on Al_x_Ga_1−x_N/GaN structures in the context of their application in microelectronic power devices and (ii) intrinsic Al_2_O_3_ films on AlN epitaxial layers grown by high-temperature oxidation for nanostructured technology of various gas/ion sensors. Particular attention is given to AES depth profiling across complete multilayer cross-sections, combining qualitative analysis of spectral line shape and intensity evolution as well as kinetic energy shifts with quantitative elemental depth distributions. This approach enables identification of chemical states and oxidation-related transformations at dielectric/semiconductor interfaces. Reported results demonstrate that AES provides micro- to nanometer-scale chemical information essential for distinguishing interfacial from the bulk properties. The capabilities and inherent limitations of AES depth profiling, including sputter-induced artifacts are also addressed, highlighting the role of optimized experimental conditions in reliable interface analysis.

## 1. Introduction

GaN-based devices form the foundation of advanced micro-/opto-electronic devices and various sensors. In particular, heteroepitaxial AlGaN/GaN structures are used in high-power and high-frequency electronics. Their exceptional properties, including a wide bandgap, strong polarization effects, and the ability to generate a high-density Two-Dimensional Electron Gas (2DEG), make them the material of choice for High Electron Mobility Transistors (HEMTs), used in satellite communication systems, radar technologies, power converters, automotive applications such as hybrid and electric vehicles and next-generation integrated photonics platforms [1,2,3,4,5,6,7,8,9,10]. However, fully exploiting the potential of these structures requires not only precise control over epitaxial growth but also careful engineering and stabilization of the surface of the AlGaN barrier and its interface with the dielectric passivation layer. In typical AlGaN/GaN-based device architectures, the dielectric layer is forming a critical boundary between the semiconductor and the gate metal.

Its primary role is to suppress the gate leakage current and improve the long-term stability of the device under high electric field operation. Effective passivation substantially reduces gate leakage within the structure [11,12], enables effective gate control by maintaining consistent threshold voltage V_TH_ under various operating and biasing conditions [13,14,15], and stabilizes device operation through precise control of interface trapping states at the gate dielectric/AlGaN interface [1,13,14,16,17,18]. These effects also contribute to suppression of dynamic ON-resistance degradation (R_ON_) and current collapse phenomenon, observed as a decrease of the drain current under pulsed operation compared with the static characteristics [12,13,14]. All these unfavorable phenomena are related to carrier trapping at the levels located both at the dielectric/AlGaN interface and near the interface on the dielectric material side. The trapped charges modify the electric field distribution in the access regions and reduces channel conductivity. Furthermore, advanced passivation layer fabrication methods, ensure minimal barrier surface damage, which is essential to maintain the integrity of the internal polarization field and a stable concentration of two-dimensional electron gas (2DEG) within the structure [14,19]. Consequently, controlling the passivation process and, thus, the concentration and distribution of trap levels in the passivated AlGaN/GaN structure is crucial achieving high efficiency and long-term reliability in power applications [1,14].

Methods for characterizing passivation layers in AlGaN/GaN devices are diverse, encompassing electrical, material, optical and interfacial analyses, all of which are essential for optimizing device performance and ensuring long-term stability. For the electrical techniques, key issue to study is suppression of the gate leakage current and threshold voltage (V_TH_) stability, typically extracted from transfer characteristics (I_D_-V_G_) [20,21]. The degradation of R_ON_ is assessed using pulsed I-V measurements that compare static and transient switching conditions [22,23,24]. A well-passivated device exhibits a minimal increase in R_ON_ during dynamic operation, indicating effective suppression of carrier trapping at the dielectric/semiconductor interface [12,18]. Likewise, high breakdown voltage (BV) values in metal-insulator-semiconductor (MIS) based devices reflect the robustness of the dielectric against strong electric fields and its ability to maintain surface integrity without triggering premature failure. The hysteresis analysis in I-V and C-V curves provides insights into the presence of charge trapping within the dielectric or at the interface. Significant hysteresis is often correlating with unstable operation and long-term reliability issues [11,18,25]. Complementary tests, such as gate stress or bias-temperature instability (BTI) measurements, are used to evaluate dielectric robustness under prolonged electric stress [26,27,28].

The analysis of interface states responsible for carrier trapping and fixed charge at the dielectric/semiconductor boundary is made primarily using photo-electric characterization methods. In [18,28], photoassisted C-V measurements were employed, using UV illumination with tunable photon energy to release charge from deep interface traps. This approach enables the identification of interface trap states and the determination of their energy distribution D_it_(E) over a wide energy range, allowing the detection of both donor-like interface states near the valence band and acceptor-like interface states near the conduction band. These studies are complemented by the results reported in [29], where photocapacitance techniques were applied to determine the interface state density distribution at the Al_2_O_3_/GaN and SiO_2_/AlGaN/GaN interfaces in MIS structures. The results showed that deep donor traps dominate in Al_2_O_3_/GaN, whereas SiO_2_/AlGaN/GaN exhibits a much shallower trap distribution, possibly due to structural differences at the oxide/GaN versus oxide/AlGaN interfaces. The photocapacitance method allowed precise determination of both the energetic position and the density of the interface traps, extending beyond the capabilities of standard C-V techniques.

The electrical and optical characteristics of AlGaN/GaN devices are closely linked to the chemical and structural properties of the passivation layer, such as its composition, interfacial bond quality, the presence of fixed charges or contaminants, and its stability under electrical or thermal stress [11,15,19]. Consequently, a comprehensive assessment of passivation effectiveness requires not only electrical benchmarking but also detailed interfacial and spectroscopic diagnostics. To this end, a range of complementary characterization techniques is employed to probe the material, chemical, and structural properties of passivation layers in AlGaN/GaN heterostructures. These include Atomic Force Microscopy (AFM) [2,13,14,30,31], High Resolution X-ray Diffraction (HR-XRD) [2,30,32,33], Spectroscopic Ellipsometry (SE) [32], Photoluminescence (PL) [32,34,35], Raman Spectroscopy [32], Time-of-Flight Secondary Ion Mass Spectrometry (TOF-SIMS) [33,36,37], Transmission Electron Microscopy (TEM) [1,14,38], and X-Ray Photoelectron Spectroscopy (XPS) [34,39,40].

In this review work, we focus on the characterization of micro-chemical properties of passivated GaN-related structures using scanning Auger nanoprobe based mainly on our reports [11,15,18,41,42,43]. This technique exhibits high surface sensitivity (average information depth of 3 nm) and exceptional lateral resolution (in the micro- to nanometer scale). Due to combination of Auger electron spectroscopy (AES) with ion-sputtering, i.e., systematic registration of AES spectra after subsequent sputtering cycles, this method is largely used to create depth profiles of element distributions in conducting/semiconducting samples. Such approach is especially useful for examining multilayer structures in semiconductor device manufacturing to understand how different elements are distributed within the device after various technological treatments. From the analysis of AES depth profiles, we obtained information about the chemical composition of (i) AlGaN/GaN heterojunction-based Metal-Semiconductor-Insulator (MIS) diodes passivated with different dielectric layers (SiO_2_, Al_2_O_3_ and SiN_x_) as well as (ii) high-temperature oxidation mechanism of AlN epitaxial layers. This information is important to optimize the passivation technology strongly influencing the electronic parameters and functional operation of (Al,Ga)N-based devices.

The further parts of this article are organized as follows: in Section 2, an idea of AES method is described, including comparison with other spectroscopic techniques, like XPS, Secondary Ion Mass Spectrometry (SIMS) and Energy-Dispersive X-ray Spectroscopy (EDS); in Section 3, a review of literature research is presented focused on passivated GaN-based structures in context of chemical analysis using AES; in Section 4, used apparatus and numerical procedure for AES analysis, as well as investigated samples of passivated AlGaN/GaN MIS structures and AlN layers are described, in Section 5, the representative results and discussion of AES profile measurements and analysis are shown; finally, in Section 6, conclusions of the presented studies are introduced.

## 2. Auger Electron Spectroscopy

**Figure 1:**
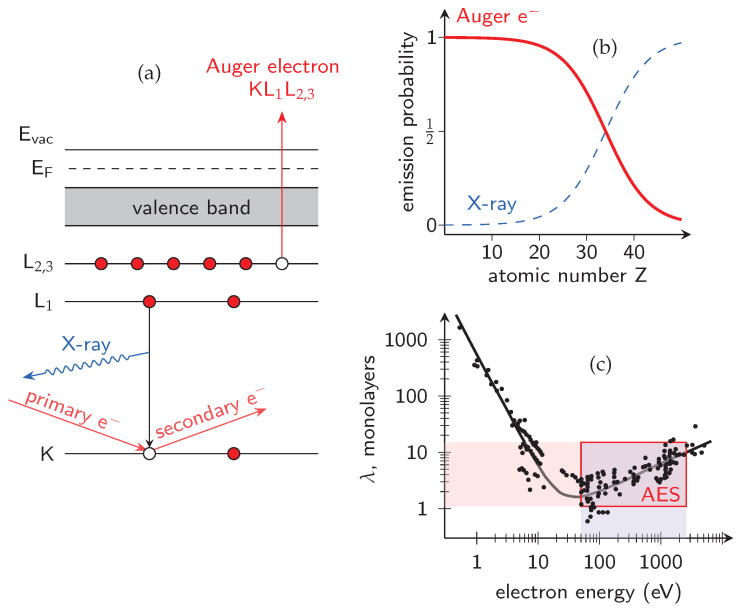
(**a**) Schematic of K-shell ionization followed by either Auger electron emission from L_2,3_ shell or X-ray photon emission (E_F_ is the Fermi level, E_vac_ is vacuum level); (**b**) emission probabilities of Auger electrons and X-rays versus atomic number Z; (**c**) electron escape depth (inelastic mean free path) versus kinetic energy, plotted using digitized data from Ref. [44].

The Auger Electron Spectroscopy (AES) is a surface-sensitive analytical technique that allows to determine the elemental composition of the top few nanometers (average information depth of 3 nm) of a material. It relies on using a high-energy (typically from 3 to 10 keV) primary electron or X-ray illumination to eject a core-level electron, which is then replaced by an electron from a higher energy level. The energy released in this process is used to eject a second, characteristic “Auger” electron or a X-ray photon, as shown in Figure 1a. The emitted Auger electron has a well-defined kinetic energy (E_k_) determined solely by the binding energy differences of the states involved, making it as a characteristic “fingerprint” of the emitting element. The measured Auger spectrum versus E_k_, which allows for the element identification in the analyzed sample, consists of the distinct Auger series, labeled according to the electron shells involved, e.g., KLL, LMM, LVV, each corresponding to a specific combination of initial and final electronic states. For example, the KLL transition involves an initial vacancy in the K shell, which is filled by an electron from the L shell, while another L-shell electron is ejected from the atom. A schematic of the KL_1_L_2,3_ process involving inner-shell transitions is illustrated in Figure 1a. It should be noted that the emission probability of Auger electrons is much larger than of X-ray photons for light elements (Figure 1b), such as Al, N, O, and Si, which play a key role in the chemistry of surface passivation of (Al, Ga)N-based structures (H and He are not detectable by AES, because of their extremely low Auger electron yields). This is because in the light elements their outer-shell electrons are less tightly bound compared to heavier elements, meaning the energy released when a higher-shell electron fills an inner-shell vacancy is often greater than the binding energy of another valence electron, causing it to be ejected as an Auger electron. In contrary, for heavier elements, this energy difference is usually smaller, making the emission of a characteristic X-ray photon more likely than ejecting a second electron (Auger electron). An advanced theoretical calculations of the emission probability for Auger electrons and X-ray photons for different elements one can find in Ref. [44].

**Figure 2:**
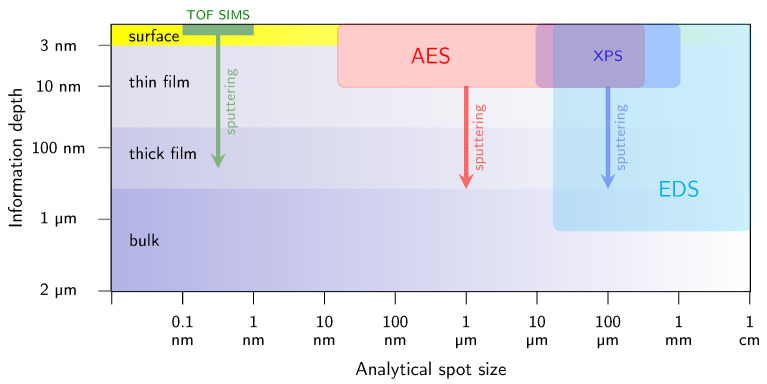
Comparison of selected methods for materials characterization in terms of their information depth and analytical spot size. AES combines the short escape depth of Auger electrons with fine lateral resolution and thus it is suitable for micro-chemical analysis of multilayer semiconductor structures.

Because the kinetic energy of the emitted Auger electrons typically spans from around one hundred to a few thousand electronvolts, their inelastic mean free path remains extremely short, namely on the order of one to several monolayers (with a pronounced minimum at tens of electronvolts), corresponding to a depth of about 0.5–10 nm, as shown in Figure 1c [45]. This shallow escape depth determines the exceptional surface sensitivity of AES and makes it suitable for semi-quantitative analysis of chemical composition and the bonding environment of solid surfaces. In this context, Figure 2 compares several material characterization methods in terms of their information depth and achievable lateral resolution. AES occupies a distinct position on this diagram due to its short escape depth and its ability to achieve a highly focused analytical spot, limited only by the size of the incident electron beam. In contrast to techniques based on electromagnetic excitation (e.g., XPS), AES employs a focused electron beam as the excitation source, allowing for lateral resolution down to tens of nanometers [46]. When combined with sequential ion sputtering, this capability enables composition depth profiling and spatially selective investigation of non-uniform surfaces, interfaces and semiconductor micro-structures, even in challenging locations such as between metal contacts in highly integrated device architectures. The combination of high lateral (minimum electron probe size of approximately 15 nm) and depth resolution (on the order of a few atomic monolayers, Figure 1c) makes AES particularly well suited for micro-chemical analysis of passivated GaN-based structures in terms of the in-depth profiles of their constituent elements, i.e., C, O, Si, N, Al and Ga. Auger measurements are typically conducted under ultra-high vacuum (UHV) conditions, i.e., at least 10^−8^ Pa, as is required by other surface-sensitive spectroscopic techniques, like XPS.

It should be enhanced that an important and unique application of scanning Auger nanoprobe is linear profiling and 2D mapping of element distributions at surfaces and interfaces [44,45]. For example, in Ref. [10] this capability was illustrated in a non-nitride system by acquiring elemental maps at successive sputter depths and assembling them into a volumetric reconstruction of the local chemical environment. Such 3D chemical imaging is able to resolve micrometer-scale lateral inhomogeneities and nanometer-scale interfacial roughness. Furthermore, the authors introduced an approach for constructing energy-dispersive Auger spectra from spatially resolved maps recorded at multiple analyzer energies. By applying linear least-squares (LLS) fitting [47], chemically distinct contributions, such as oxidized versus elemental species, could be separated and spatially localized. Such multidimensional AES approaches are particularly relevant for AlGaN/GaN structures, where even trace silicon contamination can generate lateral and vertical chemical inhomogeneities that complicate the interpretation of conventional depth profiles. Three-dimensional reconstruction and chemically resolved mapping would therefore provide a more reliable framework for distinguishing Si originating from dielectric layers, unintentional incorporation, or interface-specific processes. Despite its excellent surface sensitivity and spatial resolution, AES presents several practical limitations relevant to the analysis of passivated GaN-based structures. During measurements of dielectric films or semi-insulating layers, the sample charging should be eliminated to avoid possible shift of AES peak positions and destabilizing spectra. This is typically achieved by providing a conductive path for charge dissipation (e.g., mounting the specimen with a conductive paste or conductive underlayer) or, alternatively, by using a low-energy electron flood gun to neutralize the accumulated surface charge. The incident electron beam can also alter oxide and III-nitride surfaces by means of inducing nitrogen/oxygen losses, local oxidation, or stoichiometry changes, which may complicate interpretation of depth profiles. Quantification in AES is often affected by matrix effects, and thus multi-component systems may yield composition errors that requires cross-verification with complementary techniques. AES is generally considered limited in detecting trace impurities, and its chemical-state specificity is lower than that of XPS. However, practical sensitivity can be enhanced by optimized acquisition data and developed graphical multi-spectra analysis, as presented in Results and Discussion in Section 5. In AES depth profiling, the analytical signal originates from the topmost few nanometers of the surface exposed after each sputtering step, such that the measured composition reflects the evolving near-surface region rather than the pristine chemical state of the originally underlying layers. Reliable depth profiling by successive sputtering and analysis cycles therefore relies on minimizing beam-induced damage, primarily by reducing ion energy and ion current density, as well as by lowering the effective ion flux density through enlargement of the sputtered crater area. Within these constraints, AES depth profiling provides reliable information on relative compositional trends and interface evolution across multilayer structures.

Finally, the ion sputter-based depth profiling may introduce additional artifacts, namely ion-induced mixing, surface roughening, and preferential sputtering that blur the interface resolution; these issues are likewise encountered in XPS depth profiling. While XPS offers more reliable chemical-state information, it also suffers from charging on dielectrics, limited depth resolution, and relatively poor lateral resolution, and thus does not replace AES in interface-focused studies with high spatial resolution. In conclusion, even if the above issues should be carefully considered in the AES analysis, this technique remains a powerful tool for the micro-chemical probing the near-surface region of multilayer structures, including MIS/HEMT devices containing passivated surfaces and AlGaN/GaN heterojunctions.

## 3. AES in Characterization of (Al,Ga)N-Based Systems—A Review

The AES technique combined with ion depth profiling, although not routinely employed in the characterization of GaN-based structures, demonstrated significant analytical value in studies of surface and interface chemistry, particularly in contexts where device operation is affected by changes in charge distribution and/or contamination, such as in passivated structures or dielectric-covered regions.

In this Section, we provide a consolidated review of how AES was used to investigate the dielectric/AlGaN interface and related materials, in terms of the dielectric bulk, plasma-processed surfaces, and lateral non-uniformities, highlighting correlations between chemistry and electronic properties observed in AlGaN/GaN devices. A number of studies were focused on understanding how the chemical contamination at the dielectric/AlGaN interface influences charge trapping and current collapse. Early work demonstrated that residual carbon or oxygen at the interface can contribute to undesirable transient effects in AlGaN/GaN HEMTs, including shifts in the threshold voltage and enhanced hysteresis [12]. The improved surface preparation prior to dielectric deposition, such as plasma descum followed by HCl cleaning, was shown to significantly reduce the amount of residual carbon detectable by AES. This reduction correlated with a measurable mitigation of the current collapse, underscoring the sensitivity of device performance to even small variations in the interface chemistry. It is also well established that even trace amounts of silicon can significantly modify the surface morphology and defect density of GaN, increasing rms roughness, promoting three-dimensional growth modes and reducing carrier mobility [48]. Such Si-induced morphological and electronic modifications provide important context when interpreting Si-related features in AES depth profiles, as they may originate not only from the dielectric but also from unintentional incorporation or surface-mediated processes. Additional AES studies [11] have revealed that high-quality dielectric stacks such as SiO_2_/Si_3_N_4_ or SiN_x_/Si_3_N_4_ can exhibit very low interfacial contamination levels, with AES depth profiles showing no significant concentration of C or O at the dielectric/AlGaN boundary. In addition, electrical measurements on these structures showed a minimal hysteresis of C–V curves. The above observations prove the usefulness of AES in controlling the impurity accumulation at the dielectric/AlGaN interface. However, the direct correlation between the interface chemical and electrical properties of AlGaN/GaN structures needs more systematic studies.

To facilitate comparison among different passivation materials, typical literature ranges of D_it_ for dielectric/AlGaN interfaces are summarized in Table 1. Although D_it_ is strongly dependent on the deposition method, plasma chemistry and surface preparation, most dielectrics exhibit order-of-magnitude values of $10^{12}$–$10^{13}$ cm^−2^eV^−1^, with SiN_x_ commonly showing higher densities. These values provide a useful benchmark for relating dielectric chemistry to charge trapping and interface-related behavior observed in AES studies.

Although contamination can strongly influence the AlGaN/GaN MIS device operation, several works indicate [49] that in many cases trap-related instabilities arise predominantly from intrinsic features of the AlGaN/GaN heterostructure, like dielectric/AlGaN interface defect levels which can interact with free electrons in the 2DEG channel, rather than from interfacial impurities of trace concentrations. Such findings need both electrical measurements and chemical analysis of element distribution in AlGaN/GaN systems. In Refs. [18,25] our group reported the results of AES depth profiling combined with the photo-assisted electrical studies of AlGaN/GaN MIS devices. The AES results demonstrated the absence of O or C impurity accumulation at the dielectric/AlGaN interfaces whereas the electric characteristics, like the threshold voltage (V_th_) of capacitance-voltage (C-V) curves clearly indicated the influence of interface states. The energy distribution of the interface state density D_it_(E) observed in these structures was consistent with the Disorder-Induced Gap States (DIGS) model [50]. Within this model, the interface states are induced by the disorder of bonds near the interface produced during its formation process, rather than by impurity presence. Our further studies of dielectric/AlGaN interfaces revealed also the intrinsic origin of the fixed charge Q_f_ at these interfaces [15]. Namely, we showed that the interface positive Q_f_ depends strongly on the Al content in the Al_x_Ga_1−x_N barrier. The higher Al concentration resulted in the reduced Q_f_, in line with expectations based on the spontaneous and piezoelectric polarization. Therefore, AES serves as a tool not only for verifying the chemical cleanliness of interfaces but also for supporting models, in which the electronic interface behavior is governed by the fundamental material properties.

**Table 1:** Materials parameters of dielectrics used for passivation of AlGaN/GaN MIS devices.

Material	Type	κ	BV (MV/cm)	λ (W/m·K)	Bandgap (eV)	Key Benefits	Deposition Method	Source
SiO_2_	reference	3.9	10–12	1.4	8.9–9.0	chemically stable; very low leakage; well-understood interface	PECVD, LPCVD, ECR-CVD	[1,2,18,30]
Al_2_O_3_	high-κ	9–11	7–10	∼30	7.0–8.8	low leakage; stable interface with AlGaN/GaN; widely used baseline dielectric	ALD	[1,14,16,51,52,53,54,55,56]
SiN_x_	standard	6–8	10; 4.2	20–30	4.6–5.5	industry standard; good passivation; increases 2DEG density due to positive charge	PECVD, ECR-CVD	[14,18,51,57,58]
AlN	special	8.5–9.1	∼12	285	6.0–6.2	high thermal conductivity, structural match to GaN/AlGaN	MOCVD, sputtering	[1,51,59,60]
AlGaN diel.	III-N comp.	n/a	n/a	n/a	n/a	band/polarization engineering, epitaxial compatibility	MOCVD	[61]
AlGaN ox.	oxide	n/a	n/a	n/a	n/a	native oxidation layer; may introduce traps at interface	air-exposure	[62]
AlON	mixed	n/a	n/a	n/a	n/a	intermediate between Al_2_O_3_ and SiON; good stability; tunable band alignment	ALD	[1]
HfO_2_	high-κ	20–25	5–7	∼1	5.3–5.8	very high κ; good scaling; moderate thermal stability	ALD, MOCVD	[1]
SiON	mixed	4–6	∼9	∼10	4.5–6.0	reduced fixed charge; tunable composition; low leakage when O > 3%	PECVD	[1,51]
Ga_2_O_3_	oxide	10–11	∼8	13–27	4.4	wide bandgap; stable oxide of GaN; moderate permittivity	PLD, MBE, MOCVD	[63,64,65]
ITO	oxide	n/a	n/a	5.1	n/a	transparent conductor; useful in optoelectronics; sometimes as passivation	RF sputtering	[30]
Sc_2_O_3_	oxide	14	0.6	∼17	6.3	good therm. stab.; lattice mismatch to GaN ∼9.2%; as cap layer to stabilize MgO and MgCaO	MBE, e-beam evap.	[65,66]
MgO	oxide	9.8	n/a	n/a	8.0	unstable in humid air; lattice mismatch with GaN ∼–6.5%	PLD, MBE, e-beam evap.	[65]
MgCaO	ternary ox.	∼9–10	n/a	n/a	∼8.0	reduces mismatch (down to ∼–2%); Sc_2_O_3_ capping layer improves thermal/environmental stability	e-beam evap.	[65]

Beyond the identifying elements at the dielectric/barrier interface, AES proved highly informative in evaluating the bulk stoichiometry of dielectric layers deposited on AlGaN. In many device structures, the dielectric bulk strongly influences the fixed charge, interfacial stress, and the stability of threshold voltage. In Ref. [51], the AES depth profiling was applied to investigate grown SiN_x_, SiON, and Al_2_O__2__ dielectric films, offering detailed information on their elemental composition and chemical interfacial transitions. The results revealed that the nominal SiN_x_ layers often contain substantial oxygen content, forming oxygen-rich SiON-like material. Although the oxygen concentration at the dielectric/AlGaN interface was comparable across the different films, the oxygen incorporated into the dielectric bulk significantly altered its mechanical properties, softening the layer and reducing the interfacial stress transmitted to the AlGaN barrier. This led to the measurable differences in polarization-induced fixed charge and threshold voltage stability. It was demonstrated that, in contrast to the SiN_x_ and Al_2_O_3_ films, with the use of SiON (oxygen concentration ≥3%) in the dielectric/AlGaN/GaN system, a negative fixed charge was generated. In conclusion, the above report illustrates the value of AES in correlating dielectric stoichiometry with electrical performance.

In Ref. [67], AES was successfully used to analyze the chemical state of plasma-etched AlGaN surfaces. Combined with shallow ∼30 nm near near-surface sputter profiling (30 nm), it was shown that the plasma gas chemistry strongly affects the surface composition and the persistence of oxygen-related etch residues. Minimizing oxygen incorporation during plasma etching was found to be critical for producing smooth AlGaN surfaces and for reliable surface preparation prior to subsequent dielectric deposition and device processing. The concentration of oxygen remaining on the surface varied strongly with the gas mixture: BCl_3_/Cl_2_ resulted in considerably lower oxygen levels compared to H_2_/Cl_2_ or Ar/Cl_2_. These differences affected the formation of native Al_2_O_3_-like layers, which in turn modified the etching behavior and contributed to surface roughening.

The enhanced ability of AES to resolve lateral variations in the surface composition was demonstrated through its combination with low-energy electron-induced luminescence (LEEN) [68]. Applied to an AlGaN/GaN wafer, this approach revealed strong inhomogeneities in luminescence signals linked to both the energy band-gap edge and deep-level related emissions, suggesting local variations in the defect density and carrier concentration. The correlated AES mapping showed a pronounced variation in the N:Ga signal ratio, increasing from approximately 0.6 at the wafer center to about 1.35 near the edge, reflecting a cation composition gradient in the AlGaN barrier. The regions with higher N:Ga ratios (Al-rich or Ga-deficient) exhibited stronger defect-related luminescence and reduced conduction, while Ga-richer areas showed improved transport properties and less luminescence from deep states. These lateral composition variations were attributed to radial temperature gradients during epitaxial growth, leading to locally Ga-rich regions near the wafer center and relatively Al-rich regions toward the edge [68].

Additional insight into chemical abruptness at the AlGaN/GaN interface was provided by the AES depth profiling [69]. The measurements across the same wafer revealed local variability in the interface sharpness: while some areas displayed abrupt transitions within 1 nm, others showed gradual intermixing of Al and Ga over depths up to 10 nm. These differences were correlated with electrical and optical signatures, namely the sharper interfaces supported stronger 2DEG formation and lower defect emission, whereas more diffuse interfaces led to the weaker conduction and enhanced deep-level luminescence. In AlGaN/GaN heterostructures, the 2DEG density is governed by the polarization-induced electric field at the interface; a chemically and structurally abrupt interface enhances this confinement, which explains why sharper interfaces support stronger 2DEG formation. Taken together, these studies illustrate how AES, when combined with complementary techniques, enables nanoscale correlation between the chemical composition and device-relevant properties.

In addition to its widespread use in compositional depth profiling, AES also demonstrated to be sensitive to local electrostatic properties of GaN-based structures. In particular, spatially resolved AES studies showed that systematic shifts of Auger peak energies can be correlated with variations in local work function, surface potential, and polarization-induced band bending in laterally non-uniform GaN systems [70]. Such approaches provide an important complementary perspective by establishing the electrostatic boundary conditions of the surface prior to various technological treatments. For dielectric/AlGaN/GaN heterostructures, this sensitivity is important because polarization charge, fixed charge in the dielectric, and interface states together shape the near-surface potential that controls carrier trapping and threshold voltage stability.

AES therefore became an important complement technique to electrical analysis in studies of dielectric/AlGaN interfaces formed by the most widely used passivation layers—SiO_2_, Al_2_O_3_ and SiN_x_, which are the main subject of the presented paper. Each of these dielectrics interacts with AlGaN in a distinct way, and many of these differences have a chemical origin that can only be resolved through depth-profiling. A comparative summary of commonly used dielectric materials, their key parameters and deposition methods is provided in Table 1.

Among the commonly used dielectrics, SiO_2_ has a well-documented history of interaction with AlGaN surfaces, and its behavior provides a useful reference point for interpreting the AES profiles. A SiO_2_/AlGaN interfaces typically exhibit relatively high interface-state densities and a substantial fixed charge. Numerous studies of PECVD-grown SiO_2_ reported pronounced frequency dispersion and stretch-out in C–V characteristics, with interface-state densities in the $10^{12}$–$10^{13}$ eV^−1^cm^−2^ range and a tendency toward negative fixed charge [71,72,73]. Microscopic investigations showed that plasma-assisted deposition readily forms Ga–O, Al–O and mixed Si–O–Ga bonds at the interface, accompanied by a disordered oxynitride transition region (Ref. [49] and references cited therein). These structural features are believed to underpin the non-ideal electrical response of SiO_2_/AlGaN systems and explain why SiO_2_ generally performs less favorably than SiN_x_ or Al_2_O_3_ in terms of interface-state density and C–V stability [29,74]. Nevertheless, SiO_2_ remains technologically attractive due to its wide bandgap, large conduction-band offset, and processing flexibility, and even imperfect SiO_2_ layers can effectively suppress current collapse and reduce surface leakage [11,75]. This established literature picture provides the context for chemical analysis by AES of the PECVD SiO_2_/AlGaN interfaces studied in the present paper.

Al_2_O_3_ represents the next major dielectric system studied on AlGaN/GaN, and its interface properties where examined extensively across different deposition techniques. Numerous studies showed that Al_2_O_3_/AlGaN interfaces host a considerable density of interface states and a substantial positive fixed charge. Capacitance–voltage and photo-assisted spectroscopies consistently revealed a U-shaped distribution of D_it_(E), with the interface-state density increasing toward both band edges [18]. This behavior agrees with the disorder-induced gap states (DIGS) model [50], indicating that structural disorder and polarization-related mechanisms, rather than chemical defects, dominate the electronic response. A pronounced positive fixed charge was reported across a range of AlGaN compositions and deposition techniques, compensating the negative polarization charge in the barrier and significantly affecting the 2DEG density [18,51]. AES depth profiling showed that Al_2_O_3_ can form an abrupt contact with AlGaN without generating an additional interfacial oxide layer and without carbon accumulation [18], indicating that chemical purity alone does not eliminate interface-related electronic states. More detailed analyses identified donor-like states, located within only a few nanometers of the interface [25]. Recent studies further emphasized the role of interfacial bonding configurations, strain fields and local structural disorder in shaping both the interface-state distribution and the evolution of positive fixed charge under bias stress [20,21,76]. Collectively, the literature portrays Al_2_O_3_/AlGaN as a chemically clean but electronically complex interface, where polarization-driven charge and disorder close to the oxide/nitride boundary govern the observed electrical behavior. AES is particularly valuable in this system because it directly confirms the chemical abruptness of the interface, enabling the electronic effects to be attributed to structural and polarization-related mechanisms rather than to interfacial reactions.

In comparison with other dielectrics such as Al_2_O_3_ and SiO_2_, SiN_x_ was repeatedly shown to be particularly effective as a passivation layer for AlGaN/GaN structures. Several studies demonstrated that SiN_x_ suppresses surface states responsible for current collapse, thereby stabilizing the AlGaN surface and maintaining a higher 2DEG density, which is especially important for devices with thin or high-Al-content barriers [58]. In addition, the SiN_x_/AlGaN interface typically exhibits a pronounced positive fixed charge, and SiN_x_ is consistently reported to generate some of the highest interfacial charge densities among commonly used dielectrics [15,51,73,77]. These combined chemical and electrostatic effects explain why SiN_x_ often provides superior passivation performance in AlGaN/GaN-based devices. AES studies further showed that nominally stoichiometric SiN_x_ films often contain measurable oxygen incorporation, introduced during film growth, as demonstrated by AES depth profiling of PECVD-deposited layers, which modifies the dielectric density and interfacial stress and contributes to the variability of the resulting fixed charge [51].

AES studies further showed that nominally stoichiometric SiNx films often exhibit measurable oxygen content, which modifies the dielectric density and interfacial stress and contributes to the variability of the resulting fixed charge [51]. Together, these chemical, mechanical and electrostatic characteristics make SiN_x_/AlGaN interfaces particularly sensitive to stress transfer and oxygen contamination, both of which have been repeatedly linked to variations in interface charge and depth-profile behavior in experimental studies. Here, stress transfer refers to the mechanical coupling between the dielectric overlayer and the underlying AlGaN barrier, whereby intrinsic stresses present in the deposited dielectric film are partially transmitted to the AlGaN layer. Since AlGaN is a piezoelectric material, such stress modification directly alters the piezoelectric polarization and the associated interface charge. In oxygen-containing SiN_x_ (SiON) films, the reduced density and stiffness of the dielectric network are known to mitigate this stress transfer, thereby limiting the generation of additional piezoelectric charge [51,78,79].

Furthermore, AES was used in a number of complementary studies on AlN material and these results form an useful background for the better understanding the oxidation mechanism discussed in the next Section. Early work on AlN showed that AES can track the growth of alumina and quantify the diffusion-controlled nature of the oxidation process [80]. Thin film studies demonstrated that AES is equally effective in identifying growth modes and interfacial structure; for AlN on metallic substrates, it revealed the presence of intermediate layers and island-like morphologies that are not evident from microscopy alone [81]. In ion-modified materials, the AES depth profiling helped to determine the depth distribution of implanted nitrogen and to characterize the altered near-surface regions formed during ion bombardment [82,83]. The plasma-assisted AlN growth on silicon provided another example of how sensitive AlN is to unintentional oxygen incorporation. It was possible due to using AES which allowed to relate the source of such contamination with specific process conditions [84]. Finally, detailed analysis of the Al LVV peak demonstrated that AES can distinguish between Al–N, Al–O and metallic Al bonding environments, which is essential for reconstructing gradual chemical transitions in multilayer systems such as Al_2_O_3_/AlN/Al [85].

A detailed AES-based study of AlN oxidation was reported in Ref. [41], where the technique was applied to investigate the oxidation of AlN induced by high-temperature annealing in an oxygen atmosphere. The AES measurements enabled resolution of the gradual transformation of the AlN/Si system toward Al_2_O_3_ (alumina) through intermediate oxygen-containing Al–O–N configurations. These results highlight the sensitivity of AES to early oxidation stages and to subtle changes in local interatomic bondings, which makes the method particularly well suited for analyzing the oxidation pathways discussed in Section 5.4. It should be noted that alumina-based structures are largely used in humidity/ion sensors operating on resistance/capacitance changes, implemented in food industry.

## 4. Experimental

**Figure 3:**
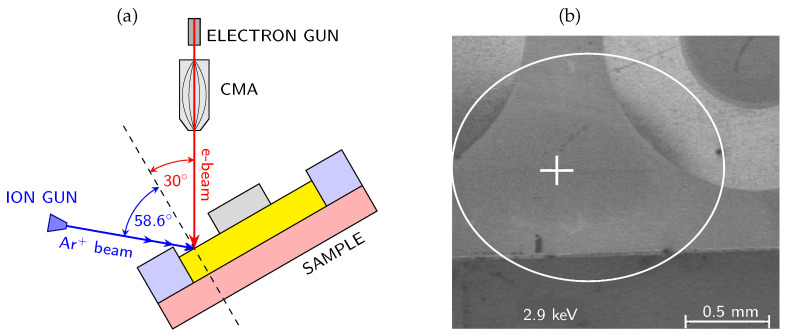
(**a**) Schematic of the geometry of measurements of AES spectra (vertical coaxial CMA and electron gun) combined with ion sputtering; (**b**) SEM image of the analyzed area on the passivated AlGaN/GaN structure with visible MIS devices (circular contacts); the point of analysis lies between devices.

In the following Sections, we focused on the overview description and discussion of results of our studies focused on the passivated AlGaN/GaN-based MIS devices and oxidized AlN layers, in which AES was used as an important technique combined with electrical and structural characterization in order to investigate the passivation film and interface chemistry, interface states and interface charge behavior [15,18,78]. The combined use of AES depth profiling and electrical measurements such as capacitance-voltage (C-V) and current-voltage (I-V) enabled a detailed interpretation of material properties at the nanoscale, including the role of dielectric composition, impurities, and mechanism of oxidation process. We would like to point out a distinctive advantage of the AES technique that is particularly relevant for the present study, namely its high lateral resolution, which enables localized chemical analysis on the micro- and nanometer scale. Thanks to the high lateral resolution, AES can be applied to structures with small dimensions, enabling depth profiling to be carried out at precisely defined locations (using secondary electron microscopy SEM) within the chosen device regions. As a result, depth profiling can be performed within laterally confined areas, such as regions between metal contacts, even on fully processed devices. This capability allows ex-situ chemical analysis of devices previously characterized by electrical methods, making it possible to directly correlate local chemical composition with electrical behavior without the need for large-area sample preparation. Figure 3 presents the schematic energy band alignment of the studied AlGaN/GaN MIS structures along with a representative SEM image acquired before the AES measurement in order to select the point for analysis.

### 4.1. Apparatus

The micro-chemical characterization of GaN-based structures was performed using a scanning Auger nanoprobe manufactured by Physical Electronics, model 670. The AES analysis combined with Ar^+^ ion sputtering, aimed at obtaining high-resolution in-depth profiles of the relative concentration of elemental components and monitoring the evolution of spectral features [43,86,87].

The system was equipped with a Schottky field emission electron gun and full cylindrical mirror analyzer with a coaxial multichannel detector. The energy of the primary electron beam was 10 keV, incident at an angle of 30° with respect to the normal of the analyzed surface. The sample current was kept constant at 20 nA. The depth profiling was performed using a scanning differential Ar^+^ ion gun operating at a low energy of 500 eV, to minimize atomic intermixing and reduce artifacts induced by ion bombardment. Depending on the sputtered material, the effective sputtering rates determined from the measured layer thicknesses and sputtering times ranged from approximately 0.6 to 1.9 nm/min under these conditions. The only exception was the SiN_x_-passivated sample, for which a higher ion energy of 2 keV was applied, resulting in an increased sputtering rate of approximately 2.6 nm/min, as discussed separately in the Section 5. The ion beam impinged on the surface at an angle of 58.6° from the normal, Figure 3. The sputtering was carried out over a 1 mm × 1 mm area, while Auger spectra were collected from a much smaller internal region that typically measures several µm^2^. Owing to the use of a scanning differential ion gun, a flat-bottom sputtered crater was obtained, and all AES spectra were acquired from the central region of the crater, where the sputtering rate is spatially uniform and representative.

To achieve enhanced depth resolution, the measurements were performed in a sequential sputter–acquire mode. One sputtering cycle consisted of a defined Ar^+^ sputtering interval (typically 0.5 min), followed by the acquisition of an AES spectrum after the sputtering was paused. Such sputtering cycles are repeated throughout the depth profile, as spectra cannot be recorded while the ion beam is on. This two-step sequence ensures a controlled material removal rate and enables precise identification of chemical shifts and, on this basis, recognition of nanoscale interfacial transitions. The differentiated spectra were acquired with 0.2–1 eV energy resolution.

It is important to note that the use of low-energy Ar^+^ sputtering (500 eV), combined with short sputter–acquisition cycles, significantly suppresses ion-beam–induced artefacts, particularly preferential sputtering [88]. At such low ion energies the sputter-yield contrast between Al, Ga, N, O and Si is strongly reduced, and cumulative surface modification is minimized. Consequently, the depth profiles for different elements at interfaces do not exhibit the characteristic slope divergence or distortion that would indicate preferential removal of specific species. This ensures that the widths of the observed interfacial regions predominantly reflect the intrinsic chemical structure rather than sputtering-induced effects, which is essential for the correct interpretation of dielectric/AlGaN interface chemistry in the following sections.

### 4.2. Numerical Analysis of Measured Spectra

The quantitative compositional analysis and AES data reduction were primarily carried out using the MultiPak^®^ software package (ver. 9, Physical Electronics), which incorporates the linear least-squares (LLS) fitting algorithm described in [47]. In this method, each spectrum within a selected energy window is modeled as a linear combination of one or more characteristic line shapes automatically extracted from the data set. By operating on the full spectral shape rather than relying on the conventional peak-to-peak measurements, the LLS procedure reduces the influence of noise and sloping backgrounds, suppresses artificial intensity in signal-free regions, and enables the separation of overlapping spectral components when their pure forms occur at any point in the depth profile. This results in improved dynamic range and a more reliable reconstruction of chemical variations across interfaces.

**Figure 4:**
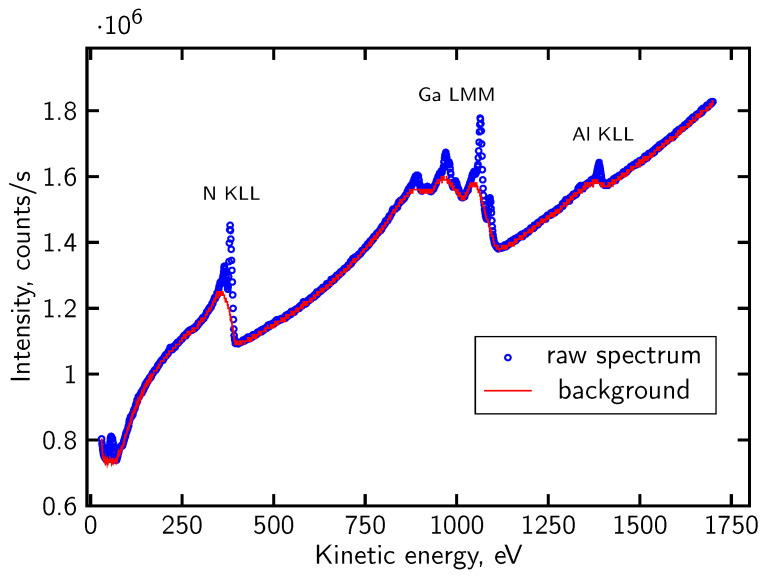
Typical registered direct AES spectrum of passivated AlGaN/GaN structure (blue dots) and theoretically determined background (red line) [47].

For certain data sets, the AES analysis was performed on direct spectra shapes evolution, following the numerical procedure described in [86]. In this approach, the background originating from scattered primary and secondary electrons is removed using a dedicated subtraction algorithm [89], Figure 4. All numerical routines implementing this procedure, including background subtraction and the subsequent peak analysis, were written by the authors. After that the Auger peaks are decomposed into their individual chemical components by fitting pseudo-Voigt functions. The fitting parameters—peak height, position, full width at half maximum and the Voigt mixing coefficient—are optimized by an evolutionary algorithm [90], which searches for the global minimum of the cost function defined for the experimental spectrum. The evolutionary-fitting framework used here was likewise implemented in author-written code. This method enables the identification of chemically shifted components, separation of overlapping contributions, and determination of peak areas used for the quantification of elemental concentrations. As demonstrated in [86], this procedure is particularly effective for analyzing passivated or multi-component oxide interfaces where chemical shifts and peak overlap play a decisive role.

Although the two numerical approaches differ in implementation, both rely on the same underlying principle: complex Auger spectra are reconstructed as a superposition of experimentally derived reference (basis) spectra. In both cases, the optimization is performed with respect to the entire spectral lineshape rather than a single characteristic energy value. As a result, not only the peak position, but also the peak width and characteristic spectral features are taken into account when separating overlapping contributions and identifying chemically shifted components. This approach is particularly important for the analysis of the N KLL region in AlGaN/GaN structures, which is a broad and structured Auger feature in which differences between chemical environments manifest primarily as changes in the overall lineshape rather than as large energy shifts.

In depth profiling techniques such as AES or SIMS, the position of an interface and the degree of interface broadening are commonly evaluated using standardized geometrical criteria [91,92,93]. The interface position is typically defined as the depth (or sputtering time) at which the signal intensity of a given layer reaches 50% of its steady-state value. The interface broadening is quantified as the depth interval over which the signal intensity changes from 84% to 16% of its maximum value. This definition corresponds to twice the standard deviation (2σ) of a Gaussian error-function profile and is widely used as a measure of the depth resolution. Conversion from sputtering time to depth is performed using the experimentally determined sputtering rates for the respective layers.

In this work, the peak positions (if given) were determined for each spectrum within the selected depth regions by statistical analysis of all spectra acquired within a given depth region. For the applied energy step (0.2 eV for direct spectra and 0.5–1 eV for differentiated spectra), the standard deviation of the mean peak position was below 1 eV.

It should be noted that, in the absence of matrix-matched calibration standards, the quantitative accuracy of AES-derived atomic concentrations is inherently limited. For standard semi-quantitative AES analysis, the typical uncertainty of atomic concentration values is on the order of 10–20%, depending on the element, matrix effects, and background subtraction procedure.

### 4.3. MIS AlGaN/GaN Structures

**Figure 5:**
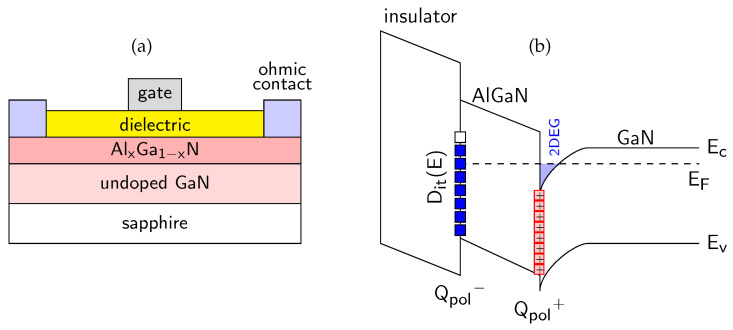
(**a**) Schematic of dielectric/Al_x_Ga_1−x_N/GaN device; (**b**) bandgap diagram of dielectric/AlGaN/GaN structure with marked valence band top (E_V_), conduction band bottom (E_C_) and Fermi level (E_F_). Full squares mean the occupied D_it_ interface states, including “frozen” states above E_F_.

**Figure 6:**
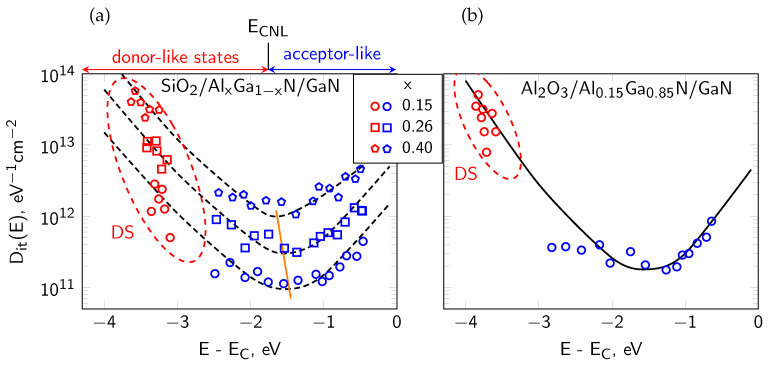
U-shaped energy distribution of the interface state density D_it_(E) at the SiO_2_/AlGaN interface (**a**) and Al_2_O_3_/AlGaN interface. Orange line indicates minima on D_it_(E) curves, DS means deep interface states close to E_v_; (**b**) as derived from the electric and photo-electric methods [18]. E_CNL_ means the charge neutrality level between the donor-like and acceptor-like interface states, according to the DIGS model of passivated III/N interfaces, after Refs. [15,18,25].

The investigated samples were AlGaN/GaN-based MIS devices passivated with three different dielectrics, i.e., Al_2_O_3_, SiO_2_ and SiN_x_ films. The materials properties of these dielectrics are summarized in Table 1. A schematic diagram of the fabricated MIS structures is shown in Figure 5a. Figure 5b presents the corresponding energy band alignment with marked polarization charge (Q_pol_^+^ and Q_pol_^−^) and interface state system D_it_(E) at the dielectric/AlGaN interface. We revealed, from our extended studies using both electric and photoelectric methods that the energy distribution of interface state density D_it_(E) exhibits a characteristic U-shape consistent with the DIGS model [18,25,50], shown in Figure 6. According to this model, the interface state system consists of both donor-like states localized in the lower part of the semiconductor band gap and acceptor-like states in the upper part. These characteristic states origin mainly of the structural disorder at the dielectric/semiconductor interface and not of the contamination atoms (like O, C). They are separated by the so-called charge neutrality level E_CNL_ with a characteristic position in the semiconductor band gap, which depends mainly on the substrate. In Figure 6a, an obvious shift of E_CNL_ versus x (toward E_V_) at the SiO_2_/Al_x_Ga_1−x_N interface agrees well with the model predictions. Furthermore, the value of D_it_ minimum increasing with x by one order of magnitude for the same dielectric layers can be related with the rising roughness of AlGaN surfaces [94], which indicates more textured, defect-prone interfaces that generally hinder device performance. In conclusion, all above observations of the interface state behavior can be well explained in terms of the DIGS model. In the Section 5, we used AES profiling to investigate the chemical properties of the passivated AlGaN interfaces with a special attention to interfacial contamination by O and C atoms, as a possible additional source of the interface states. In addition, we summarized in Table 2 the set of experimentally determined parameters of interface states at the investigated dielectric/AlGaN interfaces, as reported by our group in Refs. [15,18,78].

The passivated AlGaN/GaN were fabricated in the Research Center for Integrated Quantum Electronics, Hokkaido University, Sapporo, Japan within common research projects. The AlGaN barrier films (25 nm and 34 nm thick) on undoped GaN layers were grown by metal-organic chemical vapor deposition (MOCVD) or metalorganic vapour-phase epitaxy (MOVPE) on sapphire substrates [18]. The structures were surface-cleansed in an HF solution prior to dielectric deposition. Before dielectric film formation, in order to preserve the AlGaN surface during contact processing, the structures were protected with a 10 nm SiN layer deposited by electron cyclotron resonance chemical vapor deposition (ECR-CVD), which was later removed.

Subsequently, SiO_2_ layers (25 nm) were deposited by plasma-enhanced chemical vapor deposition (PECVD), SiN_x_ layers (22 nm) by ECR-CVD, while the Al_2_O_3_ films (20 nm) were grown by atomic layer deposition (ALD). The Al mole fractions (x) in the Al_x_Ga_1–x_N barrier layer were 0.15, 0.26, and 0.4. The thicknesses of the dielectric layers were determined by spectroscopic ellipsometry. More details on the structure technology can be found in Ref. [18].

### 4.4. Oxidized AlN Epitaxial Layers

The epitaxial AlN layers analyzed in this work were prepared and thermally oxidized with the aim of obtaining porous aluminum oxide (Al_2_O_3_) films for gas and humidity sensing applications, where a high specific surface area is a key functional requirement.

Epitaxial aluminum nitride (AlN) layers with a thickness of approximately 260 nm were grown on Si(111) substrates by metal–organic vapor phase epitaxy (MOVPE) at a growth temperature of 1060 °C. Prior to the thermal oxidation process, each sample underwent a chemical cleaning procedure aimed at removing surface contaminants and the native oxide layer.Then, the samples were subjected to high-temperature thermal oxidation at 1012 °C in a quartz reactor in a mixed (dry and wet) O_2_ + H_2_O atmosphere for oxidation times ranging from 10 to 50 min. Such approach allowed to investigate the gradual chemical and structural changes in the Al_2_O_3_/AlN/Si system with the oxidation time.

## 5. Results and Discussion

### 5.1. SiN_X_ Passivation of AlGaN/GaN

The SiN_x_/AlGaN/GaN structure analysed in this section is one of the MIS devices described above in Section 4.3, comprising an Al_0.4_Ga_0.6_N 25 nm barrier on GaN with an ECR-CVD SiN_x_ 20 nm passivation layer.

**Figure 7:**
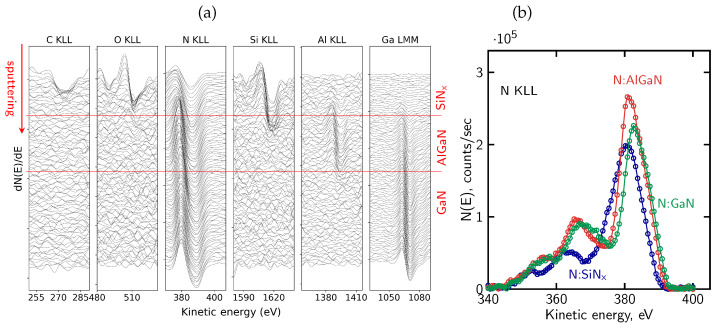
(**a**) Evolution of the differentiated Auger spectra for the main elements present in the SiN_x_ passivated structure—the red horizontal lines denote the interfaces between SiN_x_, AlGaN, and GaN layers; (**b**) representative N KLL Auger peak shapes extracted from different regions of the sample (SiN_x_, AlGaN, and GaN), illustrating distinct lineshapes associated with different bonding environments.

Figure 7a presents the evolution of differentiated AES spectra recorded during sequential ion sputtering cycles. The columns correspond to the Auger lines of all elements of the analyzed structure: C KLL, O KLL, N KLL, Si KLL, Al KLL and Ga LMM. The C KLL and O KLL signals are observed only in the first few cycles of sputtering, indicating the presence of surface contamination introduced during air exposure. Although this oxidized cap is only a few nanometers thick, it may still influence the local distribution of fixed charge or alter the effective mechanical stress within the dielectric film. It should be noted that oxygen is not a constituent element of the SiN_x_ passivation layer, and its presence is therefore attributed to surface-related contamination.

Figure 7b shows representative N KLL Auger peak shapes extracted from different regions of the sample, corresponding to the SiN_x_, AlGaN, and GaN layers. The spectra clearly differ in their overall lineshape, reflecting distinct nitrogen bonding environments in the dielectric and semiconductor regions. In particular, differences are observed not only in the peak position but also in peak width and the relative intensity distribution within the complex N KLL structure.

**Figure 8:**
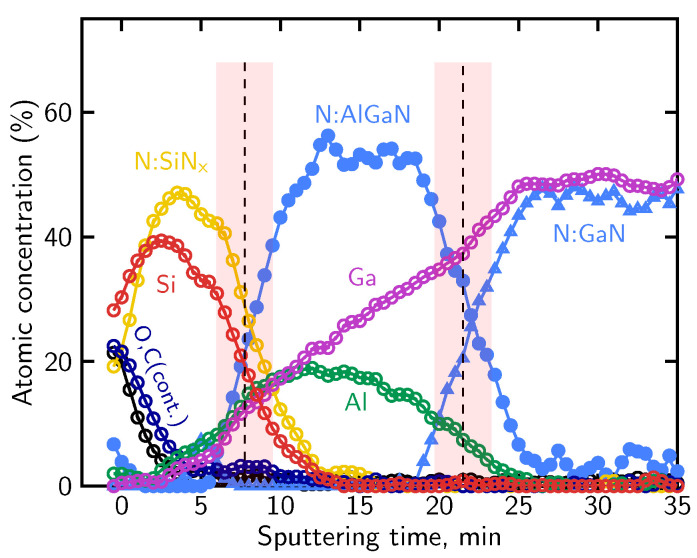
Atomic concentration profile of the SiN_x_/AlGaN/GaN sample.

The distinct N KLL lineshapes were subsequently used as reference components for the deconvolution of the evolving nitrogen signal using the LLS procedure implemented in MultiPak^®^, as described in Section 4.2. Based on this lineshape-based deconvolution, the relative contributions of nitrogen associated with the different bonding environments were determined and used to reconstruct the atomic concentration profile shown in Figure 8. The profile reproduces the expected sequence of the SiN_x_, AlGaN, and GaN layers.

A measurable oxygen contribution in SiN_x_ layers was previously reported in Ref. [51] based on AES depth profiling and associated with modified electrical behavior of AlGaN/GaN structures, and a similar oxygen-related contribution is also observed in the present measurements.

The transition between the dielectric and semiconductor regions appears broadened due to sputtering-induced artifacts. High-energy ion bombardment induces chemical state alterations and promotes knock-on effects, in which atoms from near-surface regions are displaced deeper into the structure. As a result, signals originating from surface or interfacial layers may appear artificially extended into the underlying regions of the reconstructed profile, as observed here on the dielectric/barrier interface for the Si and N signals associated with the SiN_x_ layer. Such effects are known for ion sputtering performed at high energies (>1 keV) and represent a general limitation of AES depth profiling [91,92,93]. Preferential sputtering in multicomponent systems leads to non-stoichiometric surface compositions, while atomic mixing and knock-on effects artificially broaden interfacial regions. In addition, ion-induced surface roughening further degrades depth resolution as sputtering proceeds. The combined action of these mechanisms results in broadened interfaces and extended signal tails in reconstructed concentration profiles, even for nominally abrupt heterostructures.

Using the definitions of interface position and broadening described in Section 4.1, the sputtering rate and interface broadening were evaluated for the SiN_x_/AlGaN/GaN structure sputtered at 2 keV Ar^+^ ion beam energy. The sputtering rate amounts to approximately 2.6 nm/min within the SiN_x_ passivation layer and decreases to about 1.9 nm/min in the AlGaN barrier. Based on these rates, the broadening of the SiN_x_/AlGaN interface is estimated to be on the order of 10 nm, while the AlGaN/GaN interface exhibits a broadening of approximately 8 nm. The shaded bands in Figure 8 mark the interface regions over which the interface position and broadening were determined. These values indicate a pronounced degradation of depth resolution under the applied sputtering conditions.

The nearly constant nitrogen contribution within the AlGaN region indicates a stable nitrogen bonding environment, whereas the gradual variation of the Al and Ga signals arises primarily from preferential sputtering and differing sputter yields of the group-III elements, rather than from a real change in AlGaN composition. The apparent variation of the Al and Ga signals is effectively suppressed when lower ion beam energies (e.g., 500 eV) are employed, due to a significant reduction of preferential sputtering, atomic mixing, knock-on effects, and ion-induced surface roughening, as will be demonstrated in the subsequent analyses.

### 5.2. SiO_2_ Passivation of AlGaN/GaN

SiO_2_ is commonly used as a reference dielectric in MIS structures and therefore provides a useful benchmark for comparison with other passivation layers. The SiO_2_-passivated AlGaN/GaN structure analyzed in this subsection consists of a 25 nm thick SiO_2_ dielectric layer deposited by PECVD on a 25 nm Al_0.15_Ga_0.85_N barrier grown on GaN. The sample was fabricated using the deposition and growth procedures described in Section 4.3. Depth profiling of this structure was carried out using Ar^+^ ions with an energy of 500 eV.

**Figure 9:**
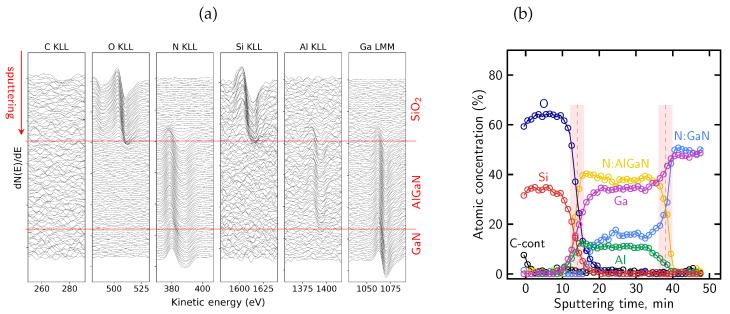
(**a**) Evolution of the differentiated Auger spectra for the main elements present in the SiO_2_/AlGaN/GaN structure, the red horizontal lines denote the interfaces between dielectric, AlGaN, and GaN layers; (**b**) corresponding relative atomic concentration profile.

In the Auger spectra shown in Figure 9a, a clear evolution of the differentiated signals corresponding to the main elements of the structure is observed during sequential sputtering. In the initial stages, the spectra are dominated by the O KLL and Si KLL signals originating from the SiO_2_ passivation layer, while contributions from the underlying semiconductor are absent. In this case, oxygen is an intrinsic constituent of the SiO_2_ dielectric layer. The O KLL and Si KLL features are characteristic of oxidized silicon species, confirming that the near-surface region corresponds to the SiO_x_ overlayer. Quantitative analysis yields an O/Si ratio of approximately 1.8, which is slightly below the stoichiometric value of 2 for ideal SiO_2_, indicating a marginally oxygen-deficient oxide. Such sub-stoichiometric compositions are commonly reported for SiO_x_ layers, where the oxygen content depends sensitively on the specific deposition parameters [95].

In contrast to the SiN_x_-passivated structure discussed above, the dielectric/AlGaN interface in the SiO_2_-passivated sample is significantly better defined. For sputtering performed at an ion energy of 500 eV, the sputtering rate within the SiO_2_ layer amounts to approximately 1.8 nm/min. Using this rate, the broadening of the SiO_2_/AlGaN interface is estimated to be on the order of 4 nm, which is substantially smaller than that observed for the SiN_x_/AlGaN interface sputtered at higher ion energy. This reduced interface broadening reflects the improved depth resolution achieved under these sputtering conditions. Within the AlGaN barrier region, both Al and Ga signals exhibit well-defined plateaus in the reconstructed concentration profile. This behavior contrasts with the SiN_x_-passivated structure discussed above, where the Al and Ga contributions changed monotonically across the barrier. The presence of stable plateaus for both group-III elements indicates a more uniform sputtering process and reflects the reduced sputtering rate achieved at the lower ion energy of 500 eV. As a result, sputtering-induced distortions such as preferential removal and atomic mixing are significantly suppressed, allowing the intrinsic compositional uniformity of the AlGaN layer to be more faithfully reproduced.

For the AlGaN barrier region, the sputtering rate amounts to approximately 1.0 nm/min. Using this rate, the broadening of the AlGaN/GaN interface is estimated to be on the order of 3 nm. This value is significantly smaller than that observed for the SiN_x_-passivated sample sputtered at higher ion energy and reflects the improved depth resolution achieved under low-energy sputtering conditions.

It should be noted that ion-induced artifacts are unavoidable in AES depth profiling, even under low-energy sputtering conditions. The use of 500 eV Ar^+^ ions reduces, but does not fully suppress, atomic mixing at the SiO_2_/AlGaN interface. A certain degree of interlayer broadening is therefore expected, particularly in the transition region where the Si and O signals decay while Al, Ga and N begin to rise. Preferential sputtering may also slightly influence the relative intensities of the elements, as oxygen is generally removed more efficiently than the metal species. These effects do not affect the qualitative reconstruction of the sequence SiO_2_ → AlGaN → GaN, and the interface width should be attributed to sputtering artifacts rather than to genuine chemical interdiffusion.

Taken together, the AES depth profiling experiment enabled unambiguous identification of the SiO_2_ film, the SiO_2_/AlGaN interface and the underlying AlGaN barrier. The oxide exhibited uniform composition across its thickness, and no evidence of oxygen or carbon accumulation at the dielectric/semiconductor boundary was detected. The AlGaN composition remained consistent with the nominal Al_0.15_Ga_0.85_N barrier. The observed interface width results from sputtering-induced mixing rather than from intrinsic chemical gradation. Overall, the PECVD SiO_2_ layer forms a chemically well-separated overlayer on AlGaN under the examined processing conditions, providing a clean reference interface for correlating the AES results with the electrical behavior of the SiO_2_/AlGaN/GaN structures discussed elsewhere in this work.

### 5.3. Al_2_O_3_ Passivation of AlGaN/GaN

The Al_2_O_3_/AlGaN/GaN sample analysed in this section corresponded to the 34 nm Al_0.26_Ga_0.74_N barrier variant introduced in Section 4.3. The structure included a 20 nm ALD-grown Al_2_O_3_ film, deposited after 10 nm SiN protection, ohmic-contact annealing and SiN chemical removal, ensuring direct comparability with the previously reported measurements [18].

**Figure 10:**
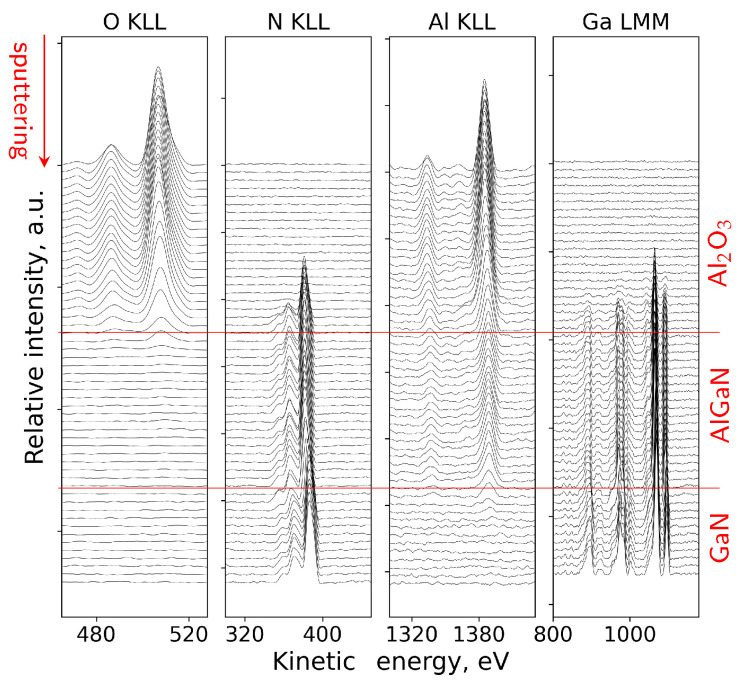
Evolution of the direct Auger N(E) spectra during sputtering of the ALD Al_2_O_3_/AlGaN/GaN sample. Red guide line indicate the approximate positions of the interfaces.

The direct spectra in this case were processed by subtracting the inelastic-electron background and by isolating the Auger signal within predefined kinetic-energy windows for the relevant transitions, as described in Section 4.2. Figure 10 shows the evolution of the direct Auger N(E) spectra recorded during sputtering. The gradual decay of the O-related contributions and the evolution of the Al, Ga and N peak shapes reflect the transition from the Al_2_O_3_ overlayer to the AlGaN barrier and finally to the GaN buffer. No abrupt distortions were observed, indicating that the sputtering conditions maintained the chemical order of the stack and that the overall layer sequence remained intact, with no evidence of artifacts beyond the expected level for the chosen ion energy.

**Figure 11:**
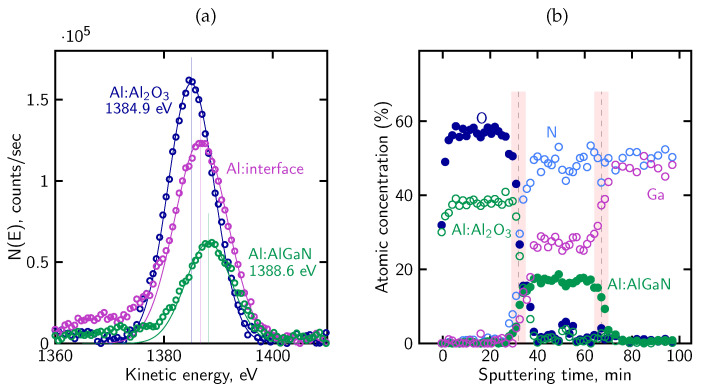
(**a**) The chemical shift of the Al KLL peak allows the aluminium signal to be resolved into contributions from Al in the oxide and Al in the AlGaN barrier; (**b**) atomic concentration profile of the Al_2_O_3_/AlGaN/GaN sample. Carbon is omitted for clarity.

In this case, the Al KLL peak showed a chemical shift of ∼3.7 eV between the Al–O and Al–N bonding environments, as shown in Figure 11a. This behavior enabled us to represent the aluminum signal as two distinct components: one associated with Al in the ALD-grown Al_2_O_3_ overlayer, and the other corresponding to Al in the AlGaN barrier. Decomposing the Al KLL line in this way made it possible to follow the dielectric film and the underlying barrier independently throughout the depth profile. As a result, the position of the Al_2_O_3_/AlGaN interface could be identified directly from the aluminum signal itself, without relying solely on the behavior of other elements.

Figure 11b shows the corresponding atomic concentration profile. The oxygen and aluminum contributions associated with the oxide decay steadily with sputtering time, while the Al, Ga and N signals from the AlGaN region rise and stabilize into a clear plateaus consistent with the nominal composition. Carbon, originating from surface contamination, was confined to the near-surface region and in this case and was omitted for clarity.

As discussed earlier, a certain degree of interface broadening is intrinsic to ion-sputtering depth profiling. Using the geometrical definitions of interface position and interface broadening described in Section 4.2, the sputtering rate and interface widths were evaluated for the Al_2_O_3_/AlGaN/GaN structure sputtered at 500 eV. The sputtering rate within the Al_2_O_3_ layer amounts to approximately 0.6 nm/min, while a comparable value of about 0.9 nm/min is obtained for the AlGaN barrier. Based on these rates, the broadening of the Al_2_O_3_/AlGaN interface is estimated to be approximately 5 nm, whereas the AlGaN/GaN interface exhibits a broadening of about 4 nm. These values indicate a well-defined interface structure and confirm the improved depth resolution achieved under low-energy sputtering conditions.

The depth-dependent evolution of the O, Al, Ga and N signals, together with the resolved position of the Al_2_O_3_/AlGaN interface, agrees with the characteristics of Al_2_O_3_/AlGaN interfaces summarized in Section 3 and provides the context needed for analyzing the electrical behavior of the corresponding MIS structures.

### 5.4. Oxidized AlN Layers

A systematic AES-based study of thermally oxidized AlN layers, covering oxidation times between 10 and 50 min, was previously reported in Ref. [41]. In that work, ex-situ AES depth profiles were acquired for a series of Al_2_O_3_/AlN/Si samples obtained at an oxidation temperature of 1012 °C in order to clarify the dynamics of the mixed oxidation process and to develop a model of oxidized AlN structures with an initial thickness of 260 nm.

Based on this experimental dataset, the present section focuses on a representative structure oxidized for 30 min, which captures the key features of the oxidation process and reflects the general trends observed across the full series. Here, we describe the corresponding AES measurement results and discuss the resulting non-linear oxidation model.

**Figure 12:**
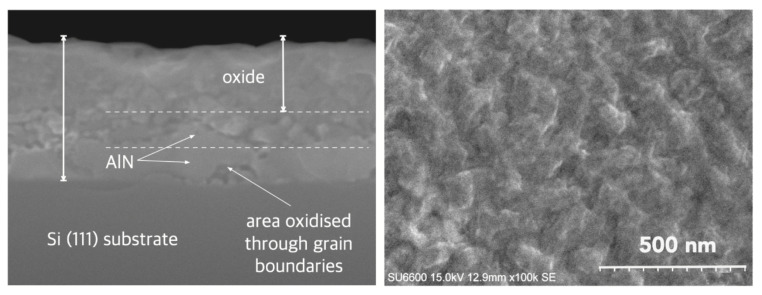
SEM images of partially oxidized AlN layer (**left**) cross-section (100 k) with visible grain morphology; and (**right**) microstructure of surface (100 k), after Ref. [41].

In Figure 12, we present the SEM images with cross-section and surface morphology of partially oxidized AlN layers. The surface microstructure shows a granular pattern with clearly defined boundaries between neighboring regions. This morphological granularity is consistent with the chemical inhomogeneity inferred from AES depth profiling, which reveals a non-uniform progression of oxidation and the formation of locally varying Al–O–N transition regions.

**Figure 13:**
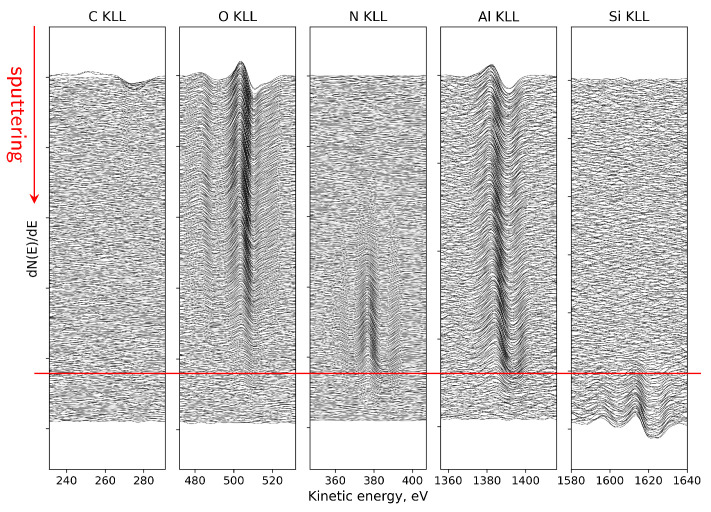
Evolution of AES spectra versus sputtering cycles for main elements present in the AlN layer after 30 min of mixed oxidation. Red guide line indicate the approximate positions of the interface between AlN and substrate.

From the visible evolution of individual Auger lines corresponding to KLL peaks of C, O, N, Al and Si registered as a function of sputtering cycles in Figure 13, one can find that (i) at the surface, the energy position of O and Al peaks can be attributed to a thin Al_2_O_3_ region, (ii) evident shift of the Al peak and weaker shift of O peak toward the higher energy as can be observed with depth where they overlap with N peak, means the reconfiguration of bonds and creation of an extended aluminum oxynitride AlON transition phase and (iii) AlN region and the sharp interface with the Si substrate (red line) are well visible.

**Figure 14:**
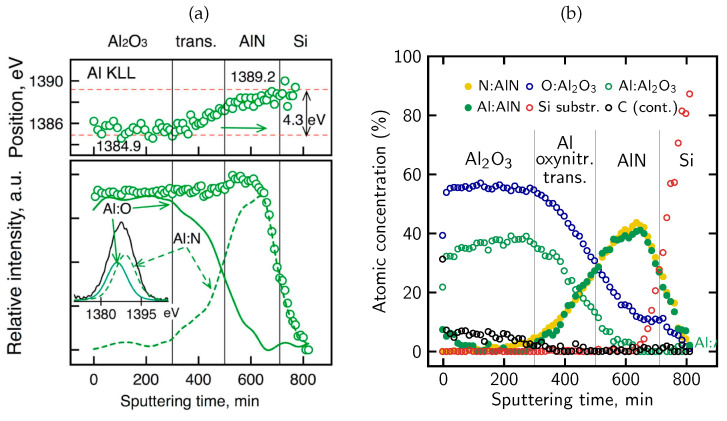
(**a**) Al KLL peak parameter changes vs. sputtering time: energy shift (by 4.3 eV) from 1384.9 eV (Al_2_O_3_) to 1389.2 eV (AlN) position (upper part) and relative peak intensity (open circles) with two components corresponding to Al:O and Al:N bonds (lower part), reproduced from Ref. [41]; (**b**) atomic relative concentration profiles (with two Al components) of Al_2_O_3_/oxynitride/AlN/Si structure after 30 min mixed oxidation.

The above observations are supported by more detailed analysis of AES KLL peak behavior, in terms of the energy position and relative intensity, as shown in Figure 14a. Namely, in the near-surface oxidized region, the Al KLL Auger peak appears at a position consistent with Al–O bonding (1384.9 eV), whereas deeper in the sample it shifts toward the value characteristic of Al–N bonds in AlN (1389.2 eV) [96,97,98]. Simultaneously, the contribution from these bonds to the overall Al peak intensity is clearly complementary. Taking into account the decomposition Al peak procedure, the AES relative atomic concentration profiles for all constituent elements (including Al components) were obtained, as presented in Figure 14b. On this basis, the ranges of individual phases were recognized and marked (vertical lines). In particular, a diffuse Al-O-N oxynitride transition region is well visible with a gradual decrease in O concentration and a simultaneous increase in N concentration with depth. In other words, the Al_2_O_3_/AlN interface is essentially a gradually evolving Al-oxynitride zone with no clearly defined boundary. The intensity of the Al KLL signal remains stable throughout the entire layer (Figure 14a).

**Figure 15:**
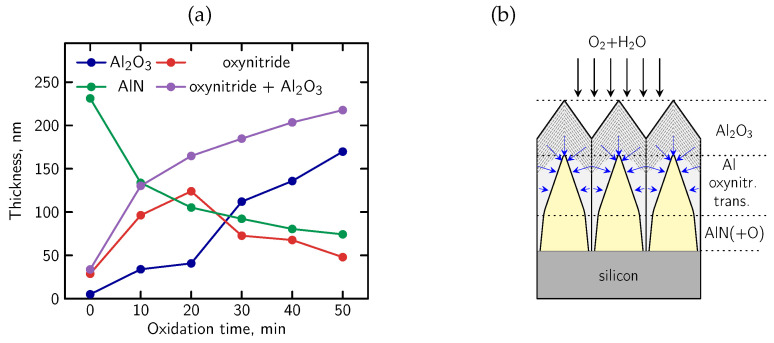
(**a**) Changes of thickness of Al_2_O_3_, Al-O-N and AlN films in Al_2_O_3_/AlN structures submitted to oxidation at different time; (**b**) proposed model of mixed oxidation process, blue arrows show the direction of oxidation on grain boundaries (see Figure 10 in [41] for details).

Finally, we analyzed the changes of the individual layer thicknesses in Al_2_O_3_/AlN structures submitted to oxidation at different time, as shown in Figure 15a. These values were estimated based on the AES profiles and ellipsometry measurements [41]. The dependencies in Figure 15a indicate that oxidation of AlN epitaxial layers is complex and consists of two competing processes, namely (i) systematic growth of the Al_2_O_3_ outer layer versus the progressive removal of N from the interface region and (ii) non-monotonic development of the oxynitride transition phase with a maximum at intermediate oxidation durations and then rapid declination. Our findings mean that the standard simplified model of a gradual planar oxidation of the whole sample is not valid. Instead, we proposed a developed model which takes into account the fact that oxidation proceeds locally—from the surface of AlN grains toward their interior—with a pronounced component perpendicular to the sample surface. The corresponding scheme of a microstructure of Al_2_O_3_/AlN/Si grain system is presented in Figure 15b. It illustrates the grain boundaries, different oxidized phases and oxygen diffusion paths (arrows) along the grains. Oxygen diffuses preferentially along grain boundaries and other microstructural defects, which act as fast diffusion pathways, while nitrogen is progressively displaced from the AlN lattice and released through the same open and interconnected paths, most likely in the gaseous phase [99]. As a result, the intermediate Al-O-N region behaves as a transient phase: it initially grows during early oxidation stages and subsequently diminishes as nitrogen is removed. It should be highlighted that the proposed grain-boundary-driven oxidation model is consistent with the observed microstructure of passivated alumina/AlN structures.

In conclusion, we developed a comprehensive chemical–morphological model of the mixed oxidation of epitaxial AlN layers based on the systematic AES profiling combined with SEM imaging which well explains the chemical evolution of alumina/AlN structures. The model assumes that oxidation initiates at the surface and grain boundaries and advances inward along them, combining vertical diffusion from the surface with lateral oxidation of individual grains. This grain-boundary controlled mechanism accounts for the absence of sharp interfaces, the gradual shifts of Auger peaks, and the evolution of the oxynitride transitional region observed in the AES depth profiles. Instead of the uniform advancing interface, each AlN grain becomes progressively oxidized inward from its periphery, at the beginning forming the transient Al-O-N phase which is gradually transformed into alumina after prolonged oxidation.

## 6. Conclusions

In our work, we focused on the analytical possibilities of the scanning Auger nanoprobe in the microchemical analysis of (i) passivated AlGaN/GaN-based structures in the context of their application in high-power devices, such as MIS-HEMTs, where the dielectric/Al_x_Ga_1–x_N interface plays a key role in device functional operation, and (ii) oxidized AlN epitaxial layers for nanostructured materials technology of various filters and gas/ion sensors. An important issue was the application of AES depth profiling to characterize interfaces created by both extrinsic (PECVD SiO_2_, ALD Al_2_O_3_, and ECR-CVD SiN_x_ films on AlGaN) and intrinsic (alumina on AlN) passivation processes.

The main aim of our studies was to identify the chemical changes through the entire multilayer sample cross-sections. The chemical AES studies were realized in two steps, namely: (i) qualitative analysis of sets of AES spectra precisely registered versus sputtering time, in terms of variations in peak intensity and shape combined with shifts on the kinetic energy axis, which allow identification of elements and their chemical bonding, and then (ii) quantitative analysis of the depth distribution of constituent elements in relative (percentage) content. On this basis, we obtained quantitative information important for understanding the oxidation processes and chemical composition of the resulting layers and interfaces.

In particular, we found that: (i) detailed chemical depth profiles of PECVD SiO_2_/AlGaN and ALD Al_2_O_3_/AlGaN structures confirmed the growth of homogeneous passivation films. Furthermore, the created insulator/barrier interfaces did not exhibit any accumulation of O and C impurities, which indicated compatibility with the disorder-induced surface state (DIGS) model of dielectric/III-nitride interfaces, and (ii) the high-temperature oxidation process of AlN layers led to the creation of two regions, namely a porous stoichiometric Al_2_O_3_ region and an Al–O–N transition one, as a result of two competing oxidation mechanisms limited by oxygen diffusion through inter-grain boundaries and through the grain bulk.

We also discussed possible artifacts during AES profiling of multilayer samples and approaches for their reduction.

We pointed out a distinctive advantage of the AES technique, particularly relevant in our studies, namely its capability to perform localized chemical analysis on fully processed devices, previously characterized mainly by electrical methods. Thanks to the high lateral resolution, AES can be applied to structures with small dimensions on the micro- and nanometer scales, enabling depth profiling to be carried out at precisely defined locations using SEM within the chosen device regions. This makes it possible to correlate chemical composition with interface electronic properties without the need for large-area sample preparation.

## Figures and Tables

**Table 2:** Reported values of interface parameters (minimum of D_it_(E), cross sections σ and fixed charge $Q_{f}$ for dielectric/AlGaN interfaces, after Refs. [15,18,78].

	$D_{\mathrm{it},\min}$, eV^−1^ · cm^-2^			σ, cm^2^			$Q_{f}$, ×10^2^ C/m^2^		
x	0.15	0.26	0.40	0.15	0.26	0.40	0.15	0.26	0.40
SiO_2_	$1\;\cdot \;10^{11}$	$5\;\cdot \;10^{11}$	$2\;\cdot \;10^{12}$	$1\;\cdot \;10^{-17}$	$5\;\cdot \;10^{-17}$	$1\;\cdot \;10^{-16}$	4.07	3.54	2.19
x	0.15	0.26	0.40	0.15	0.26	0.40	0.15	0.26	0.40
Al_2_O_3_	$3\;\cdot \;10^{11}$			$5\;\cdot \;10^{-19}÷10^{-16}$			4.07	3.54	2.19
x	0.15	0.26	0.40	0.15	0.26	0.40	0.15	0.26	0.40
SiN_x_	$6\;\cdot \;10^{11}$	$1\;\cdot \;10^{12}$	$3\;\cdot \;10^{12}$	$5\;\cdot \;10^{-19}÷10^{-16}$			3.78	2.79	1.64

## Data Availability

The data presented in this study are available on request from the corresponding author.
